# A Multicenter Randomized Bioequivalence Study of a Novel Ready-to-Use Temozolomide Oral Suspension vs. Temozolomide Capsules

**DOI:** 10.3390/pharmaceutics15122664

**Published:** 2023-11-24

**Authors:** François Ducray, Carole Ramirez, Marie Robert, Maxime Fontanilles, Charlotte Bronnimann, Olivier Chinot, Florian Estrade, Xavier Durando, Stéphanie Cartalat, Jeremy Bastid, Hugues Bienayme, Caroline Lemarchand

**Affiliations:** 1 Service de Neuro-Oncologie, Hôpital Neurologique, Hospices Civils de Lyon, Centre de Recherche en Cancérologie UMR INSERM 1052 CNRS 5286, Université Claude Bernard Lyon 1, 69008 Lyon, France; stephanie.cartalat01@chu-lyon.fr; 2 Services de Neurologie et D’oncologie Médicale, CHU et ICHUSE de Saint-Etienne, 42055 Saint-Etienne, France; carole.ramirez@chu-st-etienne.fr; 3 Institut de Cancérologie de l’Ouest, Medical Oncology, 44800 Saint Herblain, France; marie.robert@ico.unicancer.fr; 4 INSERM U1245 Unit, Cancer Centre Henri Becquerel, Université Rouen Normandie, 76038 Rouen, France; maxime.fontanilles@chb.unicancer.fr; 5 Le Havre Hospital Group, 76083 Le Havre, France; 6 CHU de Bordeaux, Service D’oncologie Médicale, Hôpital Saint André, 33075 Bordeaux, France; charlotte.bronnimann@chu-bordeaux.fr; 7 Aix-Marseille Université, Neuro-Oncology Department, APHM, CNRS, Institut de Neurophysiopathologie, CHU Timone, Service de Neuro-Oncologie, 13385 Marseille, France; olivier.chinot@ap-hm.fr; 8 Centre Eugène Marquis, 35042 Rennes, France; f.estrade@rennes.unicancer.fr; 9 INSERM U1240 IMoST, University of Clermont Auvergne, 63001 Clermont-Ferrand, France; xavier.durando@clermont.unicancer.fr; 10 UMR 501, Clinical Investigation Centre, 63011 Clermont-Ferrand, France; 11 Clinical Research and Innovation Department, Centre Jean Perrin, 63011 Clermont-Ferrand, France; 12 Oncology Department, Centre Jean Perrin, 63011 Clermont-Ferrand, France; 13 ORPHELIA Pharma, 75005 Paris, France; jeremy.bastid@orphelia-pharma.eu (J.B.); hugues.bienayme@orphelia-pharma.eu (H.B.)

**Keywords:** temozolomide, oral suspension, bioequivalence, pediatric formulation

## Abstract

Background: Temozolomide (TMZ) oral suspension (Ped-TMZ, KIZFIZO^®^) is being developed for the treatment of relapsed or refractory neuroblastoma, a rare cancer affecting infants and young children. The study assessed the safety and the bioequivalence of this novel pediatric formulation with existing TMZ oral capsules. Methods: In vitro dissolution profiles and the bioequivalence were evaluated following the European Medicines Agency “Guidelines on the investigation of Bioequivalence”. The phase I, multicenter, randomized, open-label, crossover, single-dose bioequivalence study enrolled 36 adult patients with glioblastoma multiforme or lower-grade glioma. Each patient received 200 mg/m^2^ Ped-TMZ suspension and TMZ capsules (Temodal^®^) on 2 consecutive days, with the order being randomly assigned. Fourteen blood samples were collected up to 10 h post-dosing. Bioequivalence was assessed by comparing the 90% confidence interval for the ratio of the geometric means of maximum TMZ plasma concentration (C_max_) and the area under the curve (AUCt). Other endpoints included further pharmacokinetic parameters and safety. Results: Both formulations exhibited a fast in vitro dissolution profile with more than 85% of TMZ dissolved within 15 min. For the bioequivalence study, thirty patients completed the trial as per the protocol. The ratio of Ped-TMZ/TMZ capsule geometric means (90% CI) for AUCt and C_max_ were 97.18% (95.05–99.35%) and 107.62% (98.07–118.09%), respectively, i.e., within the 80–125% bioequivalence limits. No buccal toxicity was associated with Ped-TMZ liquid formulation. Conclusions: This study showed that Ped-TMZ oral suspension and TMZ oral capsule treatment are immediate release and bioequivalent medicines. There were also no unexpected safety signals or local toxicity (funded by ORPHELIA Pharma; ClinicalTrials.gov number, NCT04467346).

## 1. Introduction

**Figure 1:**
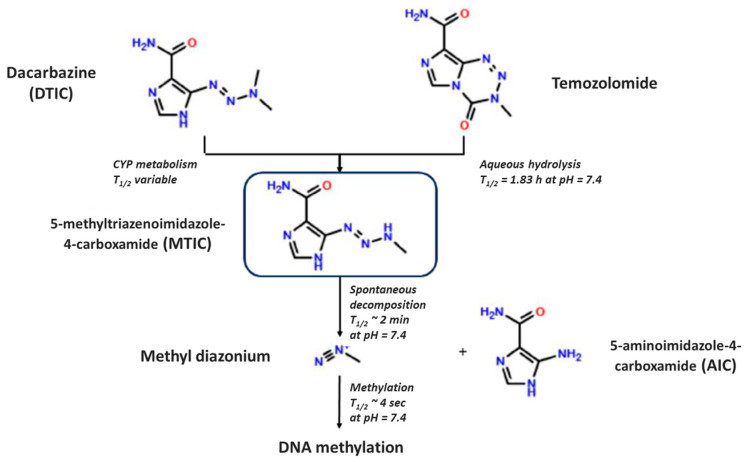
TMZ mechanism of action.

Temozolomide (TMZ) is an alkylating agent belonging to the group of triazene compounds commonly used as chemotherapeutic drugs in cancer therapy [1]. TMZ is a second generation imidazotetrazine derivative, that does not require hepatic metabolism to form the cytotoxic methylating agent, methyl triazene imidazole-4-carboxamide (MTIC), in comparison to dimethyl triazene imidazole-4-carboxamide (DTIC) [2]. TMZ undergoes spontaneous pH-dependent hydrolysis to MTIC at a physiological pH. MTIC is then hydrolyzed to the methyldiazonium cation, which is the actual methylating agent of the DNA of tumor cells, mainly at the O^6^ and N^7^ positions of guanines, and the 5-aminoimidazole-4-carboxamide (AIC), which is excreted by kidney [3]. TMZ has a half-life of 1.83 h at 37 °C in phosphate buffer (0.1 M) at pH 7.4, whereas MTIC has a half-life of approximately 2 min (min) at the same pH. There is a small pH window around the physiological pH at which the propensity of TMZ to undergo ring-opening is matched by the breakdown of the MTIC in a methylating mode [2], as shown in Figure 1. The most common techniques for the determination and quantification of TMZ in human plasma are liquid chromatography coupled with mass spectrometry (in vivo investigations) [4,5,6] and the UV spectroscopic method for analytical samples (in vitro studies) [7].

TMZ has been commercialized since 1999 under the tradename Temodal^®^ in Europe and Temodar^®^ in the US, with several oral-dose presentations: hard gel capsules 5 mg, 20 mg, 100 mg, 140 mg, 180 mg and 250 mg. TMZ is indicated for the treatment of adult patients with newly diagnosed glioblastoma multiforme and children from the age of three years, adolescents, and adult patients with recurrent malignant glioma [8]. As recommended by the International Pediatric Medical Associations, TMZ is also used off-label as a standard backbone chemotherapy for the treatment of various pediatric malignancies, notably relapsed or refractory neuroblastoma, a solid tumor affecting very young children, [9,10,11] as well as relapsed medulloblastoma [12] and rhabdomyosarcoma [13].

TMZ hard-gel capsules are not adapted for use in the pediatric population [14]. Although the intravenous (IV) dosage form was approved in 2009 with the same indications [8], the IV route increases toxicity (e.g., pain, irritation, pruritus, warmth, swelling and erythema at infusion site, petechia and hematoma) [15] and patients prefer oral chemotherapies, which are more convenient, administered at home with a good perception of efficacy [16]. As highlighted in the draft inventory of peadiatric therapeutic needs [14], an age-adapted oral TMZ formulation is of paramount importance for children suffering from neuroblastoma to ensure a good quality of life for these young patients treated in an outpatient setting and usually attending school. Facing the lack of age-adapted formulations, caregivers are instructed to open TMZ capsules and mix the contents with soft food to overcome swallowing difficulties. As TMZ is a bitter, highly toxic and unstable substance, this practice poses significant challenges to caregivers and the pediatric population. These include dose inaccuracy and drug instability [17], poor compliance, exposure to a cytotoxic drug, and environmental waste. To overcome this situation, the TMZ oral suspension (Ped-TMZ; KIZFIZO^®^) was developed as a novel pediatric oral liquid formulation that is taste-masked and ready to use. Ped-TMZ aims to prevent the inappropriate handling and exposure to toxic ingredients for parents and caregivers and is being developed for children with relapsed or refractory neuroblastoma to allow for a more precise dosage and a better compliance with treatment.

The present study aimed to evaluate the bioequivalence between Ped-TMZ and Temodal in order to assess if Ped-TMZ can be considered therapeutically equivalent to the reference oral capsule formulation. According to the EMA and FDA guidelines [18,19], with TMZ being a Biopharmaceutical Classification System (BCS) class I drug [20], a comparison of in vitro dissolution profiles can be used as a surrogate evaluation of bioequivalence. The in vitro dissolution method strategy comparing the two formulations was based on the recommendation described in the Draft Guidance on Temozolomide [21] and the FDA Guidance applied on dissolution testing [22].

As TMZ is sparingly soluble in water [23], a proportion of TMZ is already in solution in the Ped-TMZ suspension, which could potentially impact the absorption rate of TMZ (e.g., absorption from the buccal cavity) or exert formulation-specific local toxicity (e.g., mucositis). As these potential formulation-specific effects cannot be assessed in vitro, a formal bioequivalence study between Ped-TMZ and the reference product Temodal was conducted. First-in-human phase I or bioequivalence clinical studies are traditionally conducted in healthy volunteers, except for oncology drugs such as TMZ that are evaluated in cancer patients. Although the Ped-TMZ oral suspension is developed for pediatrics, the bioequivalence study was conducted in adult patients (18 years or older) with newly diagnosed glioblastoma multiforme or lower-grade glioma for ethical reasons, considering the number of blood sampling required for the pharmacokinetic (PK) analysis.

Here, we report the in vitro dissolution profiles of Ped-TMZ and Temodal capsules and the results of the formal bioequivalence study undertaken in adult patients comparing the PK parameters of both formulations after a single-dose administration. We describe the general and local safety of Ped-TMZ.

## 2. Materials and Methods

### 2.1. Products

The commercially available Temodal capsules, 100 mg, were purchased from Merck Sharp & Dohme, Puteaux, France. The investigational product Ped-TMZ, TMZ oral suspension, 40 mg/mL (brand name: KIZFIZO) was provided by ORPHELIA Pharma, Paris, France.

### 2.2. Reagents and Chemicals

For dissolution testing, analytical reagents were purified water (Milli-Q Elix Essential 5, Merck Chimie SAS, Fontenay-Sous-Bois, France) and hydrochloric acid R (37%) grade for analysis (J.T. Baker). For the bioanalytical assays, TMZ and the internal standard temozolomide-d3 (TMZ-d3) were purchased from TRC (North York, ON, Canada). The reagent-grade acetonitrile, isopropanol and methanol of gradient grade purity were purchased from Carlo Erba, trichloroacetic acid (TCA), ammonium acetate 7.5 M solution and formic acid were purchased from Sigma (St Louis, MO, USA), glacial acetic acid was from Honeywell (Charlotte, NC, USA) and dimethyl sulfoxide (DMSO) was from ACROS. Acetic acid used to prepare acetic acid 10% solution was purchased from Thermo Fisher Scientific (Waltham, MA, USA), and purified water (Milli-Q direct 8, Merck Chimie SAS, Fontenay-Sous-Bois, France) was used. Human plasma from healthy donors was provided by BioIVT (UK). Microtubes in polypropylene were purchased from Sarstedt (Nümbrecht, Germany) and Nunc™ 96 DeepWell™ Polystyrene Plates (DWP) were purchased from Thermo Fisher Scientific (Waltham, MA, USA).

### 2.3. In Vitro Dissolution Testing

The in vitro dissolution testing was performed using the basket (USP 1) apparatus (Dissolutest apparatus, Sotax, Aesch, Switzerland) described in the 10th Edition of European Pharmacopoeia 2.9.3 monograph [24] or US Pharmacopeia <711> [25] at 37 °C ± 0.5 °C and setting the agitation speed at 100 rpm. One hundred milligrams was used for the dissolution test by introducing one 100 mg TMZ capsule or 2.5 mL of Ped-TMZ (40 mg/mL) into 500 mL of dissolution medium (0.1 N HCl prepared via dilution of 8.5 mL of hydrochloric acid R (37%) to 1000.0 mL with purified water). Samples were collected using peristaltic pump (Sotax, Aesch, Swizerland) at 5, 10, 15, 20, 30 and 45 min and analyzed online by direct UV-reading after filtration on 1 µm glass filter (Macherey-Nagel, Düren, Germany). Dissolved TMZ is quantified by UV spectrophotometry (Lambda 75 UV/visible spectrophotometer Perkin Elmer, Shelton, CT, USA) at 328 nm. The evaluation is based on absorbance measurement and external standardization with relative response. The dissolution medium was used as blank solution. The assay of TMZ was validated for linearity, accuracy and precision according to the guideline ICH Q2 (R1) [26]. The method is linear over the range 20% to 100% (R = 1.0000). The accuracy and precision were tested on the three following series: 20%, 100% and 120% of TMZ. The mean recovery ranged from 99.78% to 101.4%, and all individual recovery complied with the 95.0–105.0% acceptance criteria. Regarding the precision, the coefficient of variation ranged from 0.4% to 0.7% for repeatability and from 0.7% to 1.8% for intermediate precision. The dissolution profile of TMZ was determined for both pharmaceutical forms (*n* = 12).

### 2.4. Bioequivalence Study Design and Oversight

We conducted a phase I, multicenter, randomized, open-label, crossover, single-dose bioequivalence study in seven centers in France (Hôpital Neurologique and Neurobiotec, Hospices Civils de Lyon, Lyon, France; Centre Hospitalier Universitaire de Saint-Etienne, Saint-Etienne, France; Institut de Cancérologie de l’Ouest, Medical Oncology, Saint Herblain, France; Cancer Centre Henri Becquerel, Rouen, France; Hôpital St André, Bordeaux, France; CHU Hôpital de La Timone, Marseille, France; Centre Eugène Marquis, Rennes, France; and Oncology Department, Centre Jean Perrin, Clermont-Ferrand, France).

Patient medical history was assessed and a physical examination, including buccal examination, was conducted, as detailed in the clinical study protocol (CSP). The CSP was approved by an independent ethics committee in France. The sponsor (ORPHELIA Pharma) designed the study and oversaw its conduct in collaboration with the contracted research organization, Eurofins Optimed. The study was conducted in accordance with the principles of the Declaration of Helsinki, Good Clinical Practice guidelines (ICHE6) and any relevant local regulatory requirements. Investigators were responsible for data collection and analysis.

### 2.5. Participants

Adult patients (18 years or older) with newly diagnosed glioblastoma multiforme or lower-grade glioma (grade 2 or grade 3) treated with exactly 200 mg/m^2^ TMZ as monotherapy participated in the bioequivalence study. Eligible patients had a body mass index (BMI) in the range of 18.5 to 30 kg/m^2^ and were non-pregnant and non-breast feeding. All participants provided written informed consent prior to the screening visit. Key exclusion criteria were the co-administration of sodium valproate or valproic acid (which may reduce TMZ clearance [8]) and the use of nasogastric tubes. A full list of the eligibility criteria is provided in Supplementary S1.

Patients were able to withdraw or discontinue the study if they decided to do so at any time, irrespective of the reason. A follow-up visit was planned by the investigator to conduct end-of-study visit examinations. Further criteria for withdrawal or premature discontinuation were adverse events (AEs). According to the investigator’s decision, participants experiencing AEs were monitored until conditions were resolved, or the patient was lost to follow-up.

### 2.6. Interventions

**Figure 2:**
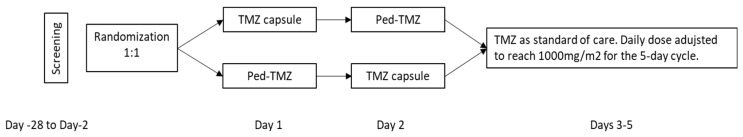
Bioequivalence study design. Screening procedures were conducted from Day 28 to Day 2 before starting study medication. Patients were treated on Day 1 and Day 2 of a treatment cycle. Patients received, under fasting conditions, 200 mg/m^2^ Ped-TMZ oral suspension on one day and 200 mg/m^2^ TMZ capsule treatment on the other day, with the order being randomly assigned in a 1:1 ratio. From D3 to D5 (outside the scope of the trial), patients received TMZ as standard of care.

Participants were randomly assigned in a 1:1 ratio, using an interactive web-based response system, to receive a single oral administration of 200 mg/m^2^ of each formulation (Ped-TMZ suspension [test product] or TMZ capsule, Temodal [reference product]) on D1 or D2 of a five-day cycle (Figure 2). No wash-out period was required between administrations, owing to the short half-life of TMZ (approximately 1.8 h [8]). Hospitalization was planned for 10 h (from 8:00 a.m. to 6:00 p.m.) for D1 and D2 or for the 48 h (h). During this period, TMZ was administered as a daily dose rounded to 300 mg or 400 mg. Prior to TMZ administration, participants were asked to maintain fasting conditions for 8 h. All patients were pre-medicated with 8 mg ondansetron (orodispersible tablet or tablet) 30 min prior to TMZ administration to prevent the nausea and vomiting associated with chemotherapy. TMZ administration took place around 8:00 a.m., after which 240 mL of tap water was used for study standardization and mouth rinsing, in a sitting position. Fasting conditions continued for 4 h post-dose, after which a standardized lunch was served. Additionally, any concomitant medications, except corticosteroid and anti-epileptic treatment, were delayed for 4 h post-dose. Concomitant treatments with valproate or valproic acid were excluded; other antiepileptic drugs were permitted.

From D3 to D5 of the treatment cycle, TMZ was administered outside of the scope of the trial as the standard of care, with the daily dose adjusted to reach a total of 1000 mg/m^2^ for the five-day treatment cycle.

### 2.7. Blood Sample Collection

Visit-specific blood handling procedures, along with clinical and biological examinations at baseline, D1, D2 and end-of study visit are detailed in the Supplementary S2. In brief, for TMZ concentration measurements, a total of 28 × 6 mL blood samples were drawn per patient (14 blood samples of 6 mL per study day). Blood samples were collected pre-dose (T0), at 10 min, 20 min, 30 min, 45 min, 1 h (h), 1.5 h, 2 h, 2.5 h, 3 h, 4 h, 6 h, 8 h and 10 h post-dose in each period (Supplementary S3). The 6 mL of venous blood was collected using a pre-chilled vacuum tube with K_2_EDTA. The blood samples were cooled in an ice-water bath immediately after sampling and centrifuged (4 °C, 2500× *g*, 5 min) to separate plasma within 30 min of blood collection.

For the determination of TMZ plasma concentrations, plasma samples were prepared in duplicate. Two aliquots of 1.0 mL of plasma sample were placed in a polypropylene tube containing 50 µL of 10% acetic acid solution (stabilizer) within 5 min after centrifugation. The acidified plasma was vortexed, and the tube was then sealed and stored frozen at −80 ± 10 °C until bioanalysis.

### 2.8. TMZ Bioanalysis

All plasma samples were prepared by protein precipitation and TMZ in human plasma extracts was assayed according to a liquid chromatography-tandem mass spectrometry (LC-MS/MS) validated analytical method.

#### 2.8.1. Sample Preparation

Plasma samples were thawed at room temperature in water batch and vortexed. Twenty (20) µL of the samples were spiked with 20 µL of Internal Standard (IS) (TMZ-d3) solution prepared in DMSO, and then, 70 µL of TCA 10% were added. The samples were vortexed for approximately 30 s and centrifuged at 20,000× *g* and 4 °C for 5 min. Twenty-five µL of supernatant were transferred into 96 DWP plates. Following addition of 400 µL of solvent A (see below the composition), plates were mixed and centrifuged at 2500× *g* and 4 °C for 5 min. The rinsing solvent consisted of a mix of acetonitrile, methanol, isopropanol and water (1/1/1/1; *v*/*v*/*v*/*v*) with 0.1% of formic acid. Then, injection of samples in LC-MS/MS was performed.

The solution of TMZ and IS (TMZ-d3) used for calibration standard and quality control (QC) are prepared in DMSO. As TMZ is unstable at physiological pH, blank matrix is prepared by acidification of plasma with acetic acid 10%. The calibration standard and QC are prepared by addition of solution of TMZ and IS to blank matrix previously centrifugated at 2500× *g* and 4 °C for 5 min.

#### 2.8.2. Chromatographic Conditions

A LC-MS API4000 (Applied Biosystems/Sciex LLC, Framingham, MA, USA) equipped with HPLC pumps LC-20AD or LC-20-ADXR (Shimadzu Corp, Japan) and an automatic temperature-controlled sampler SIL-10AC or SIL-20ACXR (Shimadzu Corp., Kyoto, Japan) was used to chromatographically separate TMZ and its Internal Standard (IS) from the plasma matrix obtained after protein precipitation. Chromatographic analysis was achieved using a Synergi Hydro-RP 2 × 100 mm, 2.5 µm column (Phenomenex, Torrance, CA, USA). The mobile phases consisted of ammonium acetate 10 mM solution containing 0.20% of formic acid (solvent A) and methanol containing 0.10% of formic acid (solvent B). The gradient elution was started with 30% of solvent B, then increased up to 95% for 0.5 min, maintained for 1.0 min, and then decreased to 30% of phase B for 0.01 min. The final composition was maintained until the end of the run (4 min). The column temperature was maintained at 40 °C throughout all measurements, whereas the sample temperature was kept at 4 °C. A volume of 5 µL sample was injected at the flow rate of 0.3 mL·min^−1^. The column eluent was directed into a triple quadrupole mass spectrometer API4000 (Applied Biosystems/Sciex LLC, Framingham, MA, USA) with an electrospray ionization source (ESI). The TMZ and TMZ-d3 were detected by monitoring mass transition using Multiple Reaction Monitoring (MRM) scan mode. The mass transitions were 198.2 > 138.3 for TMZ and 195.1 > 138.0 for TMZ-d3 with Dwell of 200 ms, the declustering potential of 60 v, the collision energy of 14 eV and a collision cell exit potential of 15 v for each transition. The data were acquired using the Analyst^®^ software (version 1.6.3, Applied Biosystems/Sciex LLC, Framingham, MA, USA).

The method was validated according to the bioanalytical method validation guidelines of the US FDA [27] and EMA [28]. The specificity of the method was demonstrated towards TMZ in presence of AIC and MTIC. The TMZ plasma assay was linear over the range of 0.1 to 25 µg/mL weighting with quadratic regression (1/x^2^). The deviation ranged from −5.88 to 10.17%. Intra-run and inter-run precision ranged from 1.01 to 9.76% and from 1.10 to 6.82%, respectively, for the 4 tested concentrations (0.1, 0.3, 12.5 and 20 µg/mL). No carry over was observed. The absence of TMZ contribution on IS and vice versa was verified. A 10-fold dilution process was validated. Bench-top and long-term stabilities at −80 ± 10 °C were validated to cover the handling and the storage of plasma samples before bioanalysis.

Finally, the robustness of the analytical method was demonstrated by sample reanalysis, with 90.15% of incurred sample reanalysis results meeting the acceptance criteria.

### 2.9. Calculation of Pharmacokinetic Parameters

The co-primary endpoints were two pharmacokinetic parameters determined from TMZ plasma concentrations obtained on D1 and D2: the observed maximum plasma concentration (C_max_) and the area under the plasma concentration curve from administration to the last quantifiable concentration at time t (AUC_t_). Secondary endpoints were the area under the plasma concentration curve extrapolated to infinity (AUC_inf_), the first time to reach C_max_ (T_max_), elimination rate constant (λ) and the plasma elimination half-life (t_1/2_). Additionally, the residual area of TMZ (%AUC_extra_) was determined from TMZ plasma concentrations obtained on D1 and D2. Safety was assessed throughout the study, including physical and buccal examination, vital signs, adverse events, concomitant treatments, electrocardiogram and laboratory examinations.

### 2.10. Statistical Analyses

Sample size calculations were based on previous TMZ bioequivalence studies [29,30,31]. It was estimated that 30 patients were required to achieve the lower limit of the 90% confidence interval (CI) and the upper limit of the 90% CI for the primary endpoints.

All randomized patients were included in the intention-to-treat set (ITTS), which provided the basis for descriptive statistics regarding quantitative and qualitative parameters. Quantitative parameters were provided using mean, standard deviation (SD), standard error of the mean (SEM), minimum, median, maximum, and number of observations, whereas qualitative parameters were provided using frequencies (n) and percent frequencies (%). Patient medical history was listed and summarized by system organ class and preferred term (Medical Dictionary for Regulatory Activity; MedDRA), if relevant. Abnormal physical findings at baseline were listed. Concomitant treatments were listed (coding performed according to the World Health Organization drug dictionary) by treatment group and patient.

Patients from the ITTS that completed the study without protocol deviations or violations thought to significantly affect the pharmacokinetic analysis (e.g., observed AEs such as vomiting and diarrhea) were included in the PK set. The pharmacokinetic and statistical analyses were carried out using Phoenix WinNonlin^TM^ software (Version 8.1; Certara, Princeton, NJ, USA) based on an independent model method (non-compartmental analysis). The primary endpoints C_max_ and AUC_t_ were analyzed using an analysis of variance (ANOVA) of two treatments, two periods and two-way crossover general linear model with the fixed effects of sequence, subject tested within sequence, period and treatment to determine error variance of ANOVA. Each ANOVA included the calculation of least-square means, adjusted differences between treatment means and the standard error associated with these differences. The level of significance for period and sequence was 0.05. However, since the difference between treatments is being tested with 90% CI, the treatment effect from ANOVA for any pharmacokinetic parameter was not considered. For the pharmacokinetic parameters C_max_ and AUC_t_, the CI for test and reference product averages were calculated using the ANOVA output from the analysis of the ln-transformed data. Inter-patient variability (%CV) was calculated for plasma concentration vs. time data and all pharmacokinetic parameters (T_max_, C_max_, AUC_t_, AUC_inf_, elimination rate constant [K_el_], t_½_ and %AUC_extra_). Intra-patient variability (%CV) was calculated for pharmacokinetic parameters C_max_ and AUC_t_ based on ln-transformed data. The ratio of geometric mean for C_max_ and AUC_t_ are reported as point estimates. The bioequivalence between the test and reference products was assessed if the 90% CI fell within the [80–125%] bioequivalence limits for C_max_ and AUC_t_ [18,32]. The assessment of bioequivalence was based on 90% CIs for the ratio of the population geometric means (test/reference) for the parameters under consideration. This method is equivalent to two one-sided tests with the null hypothesis of bio-inequivalence at the 5% significance level.

Patients from the ITTS who received at least one study treatment dose were included in the safety set for reporting safety parameters. AEs were coded according to the MedDRA. They were classified into pre-defined standard categories according to chronological criteria. Treatment-emergent AEs (TEAEs) were defined as AEs that occurred for the first time or, if present before, worsened during exposure to the drug(s). TEAEs were summarized by primary System Organ Class (SOC), Preferred Term (PT) and treatment group, with evaluations of the number of AEs and the number of patients reporting these AEs. Non-TEAEs were defined as AEs that occurred prior to study drug administration (also called “pre-dose event”) and summarized by SOC, PT, and sequence. Any potential local toxicity reaction in the mouth relating to Ped-TMZ oral suspension intake, such as but not limited to intensive local pain or ulcerative lesion of the mucosa, were categorized as AEs of special interest (AESI). The statistical analysis for safety parameters consisted of individual data listings and descriptive/inferential statistics using the SAS^®^ computer program (release 9.4, SAS Institute, Cary, NC, USA).

## 3. Results

### 3.1. In Vitro Dissolution Testing

**Figure 3:**
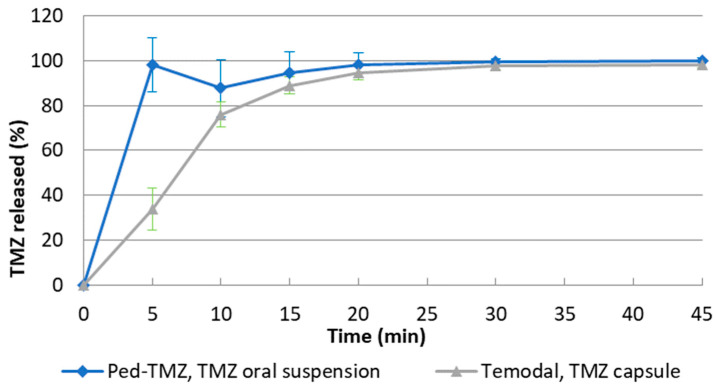
In vitro dissolution rates of Ped-TMZ oral suspension and TMZ capsules in HCl 0.1 N (*n* = 12). Error bars represent SD.

Prior to the initiation of the bioequivalence study, an in vitro dissolution study was conducted on Ped-TMZ 40 mg/mL oral suspension compared to TMZ 100 mg capsules following the EMA and FDA Guidelines [18,19]. The in vitro dissolution testing was carried out using the basket method according to a strategy based on the recommendation for the TMZ capsule described in the Draft Guidance on Temozolomide [21] and the FDA Guidance on Dissolution Testing [22]. As shown in Figure 3, 94.5% and 88.9% of TMZ is released at 15 min for Ped-TMZ and TMZ 100 mg capsules, respectively. According to the guideline, as more than 85% of TMZ is dissolved within 15 min for both products, they can be considered as immediate-release medicines and no statistical calculation is required for the demonstration of similarity according to the EMA guideline [18]. Of note, the difference between the dissolution profiles at 5 min (33.9% for Temodal compared to 98.1% for Ped-TMZ) is expected and reflects the progressive solubilization of the hard gel capsule (gelatin) of Temodal. From the 10 min dissolution timepoint, both profiles have a similar shape.

### 3.2. Pharmacokinetic Bioequivalence Study

#### 3.2.1. Baseline Demographics

**Figure 4:**
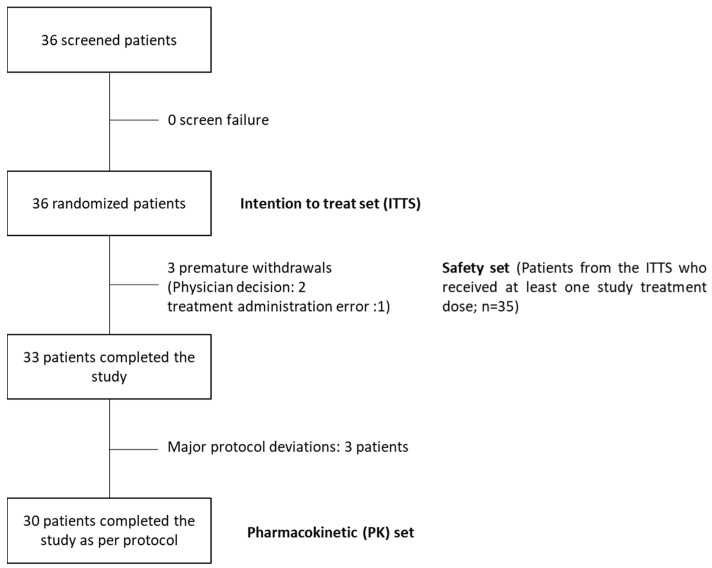
Patient disposition according to CONSORT and details of the different population sets.

From September 2020 through to December 2021, 36 patients were enrolled in the study and comprised the ITTS. Three patients were withdrawn, two upon the investigator’s decision and one due to a treatment administration error (for TMZ capsule treatment, a dose of 500 mg was administered instead of 300 mg). Four major deviations involving three additional patients were detected during the study and led to the exclusion and replacement of these patients (two missing pharmacokinetic samplings in one patient and two patients did not follow the fasting protocol). Thirty (30) patients completed the study without protocol deviations or violations (PK set). Patient disposition and details regarding the different population sets are presented in Figure 4.

Patient demographics and baseline characteristics are summarized in Table 1 and further described in Supplementary Table S1. Most participants were male (27/36, 75%), with a mean age of 52 years. Their BMIs ranged from 19.8 to 30.6 kg/m^2^. Most patients (30/36) had a Karnofsky index equal to or higher than 80. In the ITTS, the Karnofsky performance score ranged from 60 to 100%, with a mean of 85.3 ± 11.3%. Most patients (30/36, 83.3%) had at least a medical or surgical history, but this was not considered to have an impact on safety and pharmacokinetic assessment criteria. Concomitant treatments, including anti-epileptics and steroids, are described in Supplementary Table S2.

#### 3.2.2. Pharmacokinetic Parameters and Plasma Concentrations

**Figure 5:**
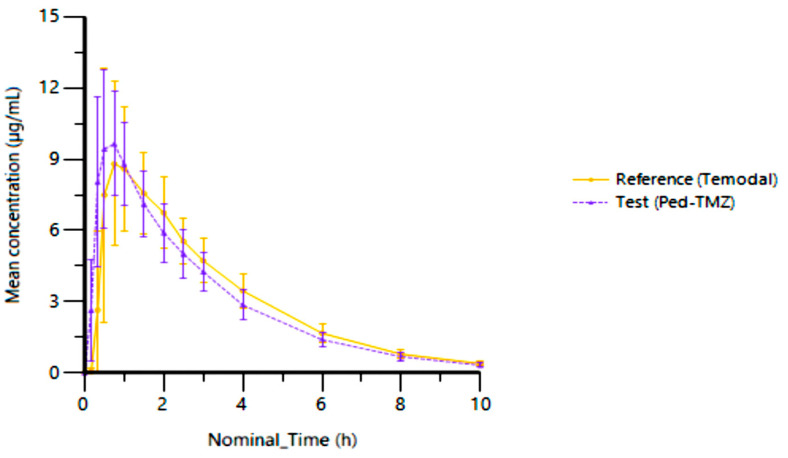
Plasma TMZ concentration after a single administration of 200 mg/m^2^ Ped-TMZ oral suspension (test) and TMZ capsule (Temodal, reference) (mean ± SD, *n* = 30 patients).

For the 30 patients of the pharmacokinetic (PK) set, 28 × 6 mL blood samples were drawn per patient (14 blood samples per day) from T0 (pre-dose) to 10 h post-dose. The mean plasma concentration–time curves are shown in Figure 5.

The PK parameters calculated following the oral administration of the TMZ capsule (reference) or Ped-TMZ oral suspension (test) are presented in Table 2. The co-primary endpoints C_max_ and AUC_t_ were similar after oral administration of the two formulations and even showed less variability after administration of the Ped-TMZ oral suspension (%CV = 23.2% and 16.2%) than after administration of the TMZ capsule treatment (%CV = 37.1% and 18.2%). The mean C_max_ (±SD) was 10.94 ± 2.54 µg/mL for Ped-TMZ and 10.51 ± 3.89 µg/mL for TMZ capsule. The mean AUC_t_ (±SD) was 30.47 ± 4.94 h·µg/mL for Ped-TMZ and 31.47 ± 5.73 h·µg/mL for the TMZ capsule treatment. Secondary endpoints (mean ± SD) for Ped-TMZ vs. TMZ capsule treatment included AUC_inf_ (31.38 ± 5.06 vs. 32.58 ± 5.84), T_max_ (0.65 ± 0.30 vs. 0.91 ± 0.41 h), K_el_ (0.37 ± 0.04 vs. 0.36 (0.03) 1/h) and t_1/2_ (1.91 ± 0.21 vs. 1.93 ± 0.16 h).

#### 3.2.3. Bioequivalence of Ped-TMZ Oral Suspension vs. TMZ Capsules

Assessment of bioequivalence was based upon the 90% two-sided CI of the geometric means ratio for the AUC_t_ and C_max_ parameters. Two products are deemed bioequivalent if the 90% two-sided CIs are within the acceptance interval (80% to 125%). The geometric means for Ped-TMZ vs. TMZ capsule treatment were 30.09 h·µg/mL vs. 30.96 h·µg/mL for AUC_t_ and 10.67 µg/mL vs. 9.92 µg/mL for C_max_, respectively (Table 3). The ratios of geometric means (Ped-TMZ/TMZ capsule) for AUC_t_ and C_max_ were 97.18% and 107.62%, respectively, and the 90% CIs for AUC_t_ (95.05–99.35%) and C_max_ (98.07–118.09%) were within the 80% to 125% limits for bioequivalence. Intra-patient variability for AUC_t_ and C_max_ were 2.53% and 4.47%, respectively. The results satisfied the bioequivalence criteria of the Bioequivalence Guidelines (90% CIs between 80% and 125%). The two examined medications Ped-TMZ oral suspension and TMZ capsules are bioequivalent.

#### 3.2.4. Safety Assessment

Among the 35 patients included in the safety set, 34 patients received at least one study treatment dose, as scheduled in the protocol. No serious adverse events (SAEs) were reported during this study. During the overall study period, five patients (14.3%) reported the occurrence of six treatment-emergent AEs (TEAEs), i.e., AEs that occurred after treatment initiation. Of these, four TEAEs were experienced by four patients after Ped-TMZ oral suspension administration. These included two episodes of headache of mild intensity, judged by the investigators to be unrelated or unlikely to be related to the study treatment; one episode of mild nausea, judged to be related to the treatment; and one episode of mild diarrhea, judged by the investigators to be unrelated to the treatment. Two TEAEs (one episode of moderate lymphopenia and one episode of headache of mild intensity) were experienced by two patients after TMZ capsule administration and were all judged to be unrelated to the treatment. Three pre-dose events, that were not TEAEs, were also reported. All the TEAEs were resolved before the end of the study. There was no evidence of clinically relevant treatment-related abnormalities for laboratory parameters, vital signs, physical findings or electrocardiogram recordings.

As Ped-TMZ is the first oral liquid dosage form of TMZ, buccal adverse events of special interest (AESIs), defined as any potential local toxicity reaction in the mouth, such as intensive local pain or ulcerative lesion of the mucosa, were carefully monitored. Importantly, no such buccal toxicity associated with the use of Ped-TMZ was detected during this study.

## 4. Discussion

TMZ is widely used as standard chemotherapy to treat a broad range of pediatric malignancies, including relapsed or refractory neuroblastoma [9,10,11], which affects very young children. However, the currently available oral dosing forms are not adapted to the pediatric population, and an age-appropriate TMZ formulation is on the priority list established by the EMA [14]. Ped-TMZ, an oral suspension of TMZ (KIZFIZO), has been developed with the aim of providing an age-appropriate formulation to the pediatric population, with accuracy, flexibility of dose adjustment according to the BSA, ease of use and taste masking.

Here, we investigated the in vitro dissolution profile as well as the pharmacokinetic properties and safety of the TMZ oral suspension (Ped-TMZ) developed to address the needs of pediatric patients. This open-label and randomized clinical study was conducted, in adult patients with gliomas for ethical reasons, to assess the bioequivalence of a single dose of 200 mg/m^2^ of TMZ administered as an oral suspension (Ped-TMZ) or capsule (Temodal) under fasting conditions. The plasma concentration–time profile and PK parameters were similar for both treatments. The 90% CIs of the geometric means of the exposure PK parameters, C_max_ and AUC_t_, were contained within the bioequivalence limits of 80% to 125%, demonstrating the bioequivalence between Ped-TMZ, TMZ oral suspension and TMZ capsules.

**Figure 6:**
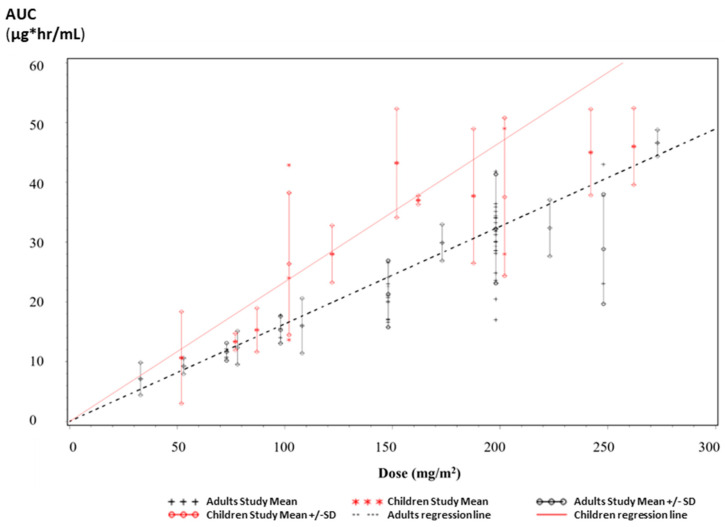
AUC from TMZ PK studies in adults and children.

**Figure 7:**
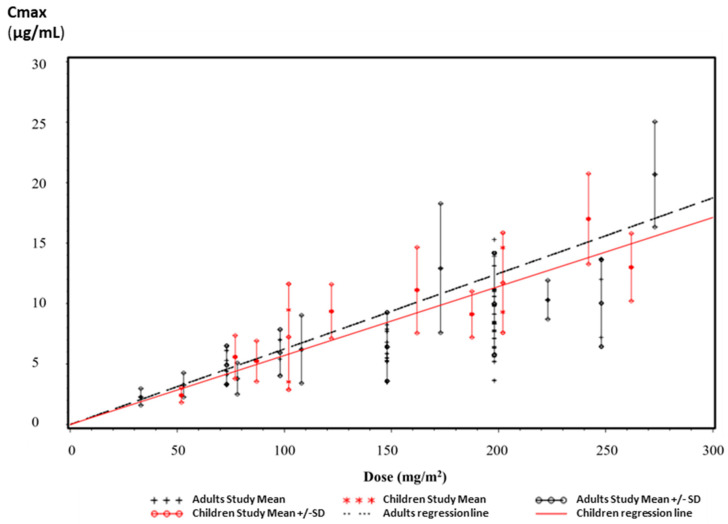
C_max_ from TMZ PK studies in adults and children.

Our PK findings (mean PK parameters) are in-line with previous PK analyses from 359 adult patients collected in three phase I studies (patients with advanced cancer without bone marrow involvement) and three phase II studies (patients with glioblastoma multiforme or anaplastic astrocytoma) as described in the Temodal European public assessment report (EPAR) [33]. Since Temodal approval, many PK clinical investigations of TMZ have been conducted, currently offering a bigger set of patients. Thirty-one (31) PK studies of TMZ in adult patients [3,6,34,35,36,37,38,39,40,41,42,43,44,45,46,47,48,49,50,51,52,53,54,55,56,57,58,59,60,61,62] and 12 PK studies conducted in children [58,63,64,65,66,67,68,69,70,71,72,73], where the AUC and C_max_ mean value are reported with or without CV, were selected for the determination of PK parameters in these two populations. Despite the limitations of pooling the data (e.g., quality not necessarily the same across all studies), the mean value of the AUC and C_max_ calculated from the pooled data of these studies were reported in the Figure 6 and Figure 7, as a function of the administrated dose in mg/m^2^. The linear regression was also calculated without intercept (i.e., the parameter is predicted to be 0 when the dose is 0).

The mean AUC value of Ped-TMZ after a dose of 200 mg/m^2^ (30.47 µg·hr/mL), which is close to the one of Temodal in our study (31.47 µg·hr/mL), is very consistent with the published data (Figure 6). For the dose of 200 mg/m^2^, AUC values collected from the literature ranged from 17.00 to 41.80 µg·hr/mL [3,44], with a mean of 32.03 µg·hr/mL from the pooled data set. Similarly, C_max_ determined for Ped-TMZ (10.94 µg/mL) and Temodal (10.51 µg/mL) are consistent with the data collected from the literature (Figure 7). C_max_ ranged from 5.20 to 15.3 µg/mL [3,35], with a mean value from the pooled data of 9.98 µg/mL for the dose of 200 mg/m^2^.

In the perspective of pediatric use of Ped-TMZ, the PK data (AUC and C_max_) from clinical studies conducted in adults was compared to those coming from clinical studies conducted in the pediatric population. This descriptive analysis interestingly highlights some differences and similarities of the main PK parameters (AUC and C_max_) when comparing the data in adults and in children. The AUC values observed in children are higher than in adults (Figure 6), which is consistent with the EPAR of Temodal [33] and the current EMA summary of product characteristics (SmPC) [8], and which is expected when dosing is carried out by BSA in children. There was no difference in C_max_ between children and adults (Figure 7), which is not in agreement with the EPAR of Temodal reporting higher C_max_ in children [33]. The ongoing TEMOkids pediatric PK population study will help to better characterize the exposure of children to Ped-TMZ.

Our investigations showed that Ped-TMZ reached T_max_ after 0.6 h, whereas the mean t_1/2_ was 1.91 h, values which are congruent with known T_max_ between 0.5 and 1.5 h and t_1/2_ of approximately 1.8 h for TMZ capsules [2,8,15].

Despite the water-soluble and immediately bioavailable fraction of TMZ (approximately 8%, data not shown) in the Ped-TMZ suspension, the C_max_ and T_max_ did not differ to the reference product Temodal. No rapid oromucosal absorption of TMZ can be suspected from the buccal cavity: the total dose of TMZ in Ped-TMZ is swallowed and absorbed from the gastrointestinal tract. The absorption PK profile is consistent with an in vitro, dissolution profile, showing the fast release of TMZ (more than 85% dissolved within 15 min). The slight difference of the TMZ release observed at the very early timepoint of the dissolution profile (close to 100% for Ped-TMZ vs. 30% for Temodal) reflects the solubilization of the hard gel capsule of Temodal and is confirmed as insignificant regarding the in vivo bioequivalence.

As a first in human trial, this study aimed to report the short-term safety profile of the Ped-TMZ oral suspension, which is in accordance with that expected for the TMZ capsule treatment [8,74]. No serious AEs were reported. A total of six TEAEs were reported including gastrointestinal disorders, blood and lymphatic system disorders or nervous system disorders. All were mild to moderate in severity and only 1/6 was considered to be related to the study drug (Ped-TMZ) by the investigator. They were resolved before the end of the study. One limitation of the present study is that it assessed the safety of Ped-TMZ for a short treatment period (only 2 days). The full safety profile of Ped-TMZ, including the evaluation of any potential oral complications following administration of Ped-TMZ in the longer term and in the targeted population (pediatrics), is being investigated in the TEMOkids trial (NCT04610736).

This study aimed at collecting any potential signal of buccal toxicity specifically related to a liquid formulation of a chemotherapeutic drug. The lack of selectivity of antineoplastic oral chemotherapy may result in direct toxicity, upon action of the drug on the oral mucosa, or indirect toxicity, as a consequence of chemotherapeutic drug-induced bone-marrow suppression or myelosuppression. Local adverse events such as oropharyngeal mucositis are not mentioned in the Temodal SmPC [8]. Areview of the literature led us to identify one study evaluating alisertib in combination with irinotecan and oral TMZ in pediatric and young adult neuroblastoma patients, in which mucositis was reported in 9% of the 244 courses [75], although most likely attributable to alisertib. Nevertheless, a liquid formulation of a cytotoxic drug such as Ped-TMZ may potentially irritate the mucosa when administered orally. Some precautions were taken with administration of 240 mL of water immediately after the treatment with Ped-TMZ. Buccal toxicity was specifically monitored during the study with buccal examination before and after administration of Ped-TMZ and at the end of the study. The first signs of oral complications (inflammatory/vascular phase) generally occur shortly after the administration of chemotherapy, with the release of epithelial cytokines producing local tissue damage that leads to early stage of mucositis [76]. In this clinical study, no acute buccal toxicity (i.e., early stage of mucositis) occurring after the administration of Ped-TMZ was detected, which is in line with the safety profile of a hospital compounded liquid formulation [77]. Further potential oral complications are being specifically monitored in the pediatric TEMOkids study, in which patients receive up to six cycles of treatment.

## 5. Conclusions

Ped-TMZ oral suspension (KIZFIZO) is the first drinkable form of TMZ specifically developed to address the needs of pediatric cancer patients or patients presenting swallowing difficulties. This study demonstrated the bioequivalence of the Ped-TMZ oral suspension vs. TMZ oral hard-gel capsules (Temodal), meaning that the two formulations are therapeutically equivalent. The safety profile of Ped-TMZ in this first in human study is similar to the one of the TMZ capsules, without any acute buccal complication occurring after the administration of Ped-TMZ; although, the short treatment duration is a limitation of the present study. The PK and safety profile of Ped-TMZ in childhood populations is currently being further evaluated in the TEMOkids trial (NCT04610736).

## Figures and Tables

**Table 1:** Baseline demographics from the ITT (*n* = 36), Safety (*n* = 35) and PK (*n* = 30) sets. BMI, body mass index; BSA, body surface area; ITT, intention-to-treat set; Max, maximum; Min, minimum; PK, pharmacokinetic set; SD, standard derivation.

	ITTS (*n* = 36)	Safety Set (*n* = 35)	PK Set (*n* = 30)
Age (years)			
Mean ± SD	52.3 ± 14.8	52.0 ± 14.9	52.8 ± 14.6
Min/Max	20/79	20/79	20/79
Sex, n (%)			
Female	9 (25.0)	9 (25.7)	8 (26.7)
Male	27 (75.0)	26 (74.3)	22 (73.3)
Height (cm)			
Mean ± SD	173.22 ± 8.00	173.11 ± 8.09	172.93 ± 7.97
Min/Max	157/188	157/188	157/188
Weight (kg)			
Mean ± SD	74.45 ± 10.96	74.38 ± 11.11	74.40 ± 11.79
Min/Max	58.0/102.0	58.0/102.0	58.0/102.0
BMI (kg/m^2^)			
Mean ± SD	24.79 ± 2.91	24.79 ± 2.95	24.82 ± 2.93
Min/Max	19.8/30.6	19.8/30.6	19.8/30.6
BSA (m^2^)			
Mean ± SD	1.89 ± 0.17	1.89 ± 0.17	1.89 ± 0.18
Min/Max	1.6/2.3	1.6/2.3	1.6/2.3

**Table 2:** Pharmacokinetic data of the pharmacokinetic set (*n* = 30 patients). Results are displayed as arithmetic mean. %AUC_extra_, percentage of extrapolated AUC; AUC_inf_, area under the plasma concentration curve extrapolated to infinity; AUC_t_, area under the plasma concentration curve from administration to the last quantifiable concentration at time t; C_max_, observed maximum plasma concentration of TMZ; CV, subject variability; K_el_, estimated by the linear regression of the logarithm of the terminal concentration as a function of time; Max, maximum; Min, minimum; n, number of patients; SD, standard derivation; t_1/2_, plasma elimination half-life, calculated as t_1/2_ = ln_2_/K_el_; T_max_, first time to reach C_max_.

Parameter (Unit)	Statistic	TMZ Capsule (Reference) (*n* = 30)	Ped-TMZ Oral Suspension (Test) (*n* = 30)
T_max_ (h)	Mean	0.909	0.649
	SD	0.405	0.302
	Median	0.77	0.63
	Min-max	0.33–2.00	0.33–1.53
C_max_ (µg/mL)	Mean	10.506	10.939
	SD	3.894	2.540
	% CV	37.1	23.2
AUC_t_ (h·µg/mL)	Mean	31.471	30.467
	SD	5.727	4.939
	% CV	18.2	16.2
AUC_inf_ (h·µg/mL)	Mean	32.584	31.376
	SD	5.840	5.062
	% CV	17.9	16.1
K_el_ (1/h)	Mean	0.362	0.367
	SD	0.031	0.035
	% CV	9.5	9.5
t_1/2_ (h)	Mean	1.928	1.909
	SD	0.163	0.205
	% CV	8.4	10.7
%AUC_extra_ (%)	Mean	3.454	2.901
	SD	0.954	0.895
	% CV	27.6	30.8

**Table 3:** Summary of pharmacokinetic parameters from the PK set (*n* = 30) treated with Ped-TMZ oral suspension and TMZ oral capsule. Results are displayed as geometric mean.; AUC_t_, area under the plasma concentration curve from administration to the last quantifiable concentration at time t; CI, confidence interval; C_max_, observed maximum plasma concentration of TMZ.

	TMZ Oral Capsule (Reference) (*n* = 30)	Ped-TMZ Oral Suspension (Test) (*n* = 30)
C_max_ (µg/mL)	9.92	10.67
C_max_ ratio (%) (90% CI)	107.62 (98.07;118.09)	
C_max_ % CV	4.47	
AUC_t_ (h·µg/mL)	30.96	30.09
AUC_t_ ratio (%) (90% CI)	97.18 (95.05;99.35)	
AUC_t_ % CV	2.53	

## Data Availability

Due to commercial restrictions, the data is not publicly available.

## Supplementary Materials

The following supporting information can be downloaded at: https://www.mdpi.com/article/10.3390/pharmaceutics15122664/s1, Table S1: Medical and surgical history from the ITTS (>10%); Table S2: Concomitant medications starting before/after TMZ administration from the ITTS (>10%) by treatment sequence and totalled over all patients; Supplementary S1: Full list of eligibility criteria; Supplementary S2. Study period flow chart; Supplementary S3. Collection of blood samples.
