# Supplementary material for: A Multicenter Randomized Bioequivalence Study of a Novel Ready-to-Use Temozolomide Oral Suspension vs. Temozolomide Capsules
Source: Pharmaceutics. 2023 Nov 24;15(12):2664. doi: 10.3390/pharmaceutics15122664 (PMC10747054; doi:10.3390/pharmaceutics15122664)
Supplement: Supplementary file 1 [file pharmaceutics-15-02664-s001.zip › Supplementary S1.pdf]

**Supplementary S1. Full list of eligibility criteria**

*1.1 Inclusion criteria*

1- Male and female patients at least 18 years of age at time of inclusion.

2- Females of childbearing potential/sexually active males with a partner of childbearing potential; commitment to consistently and correctly use an acceptable method of birth control (oral, transdermal, systemic or implant contraception birth control, intrauterine devices, diaphragm or condoms) for the duration of the trial and for 4 months after the last study drug administration; negative pregnancy test at screening.

3- Females of non-childbearing potential: either surgically sterilized or at least 1 year postmenopausal (amenorrhea for at least 12 months).

4- Patients with glioblastoma multiforme or low-grade glioma (grade 2 or 3), in need of temozolomide as monotherapy, at 200 mg/$m^2$.

5- Patients having completed at least one cycle of temozolomide without signs of hypersensitivity.

6- Patients having an Indice of Karnofsky, IK ≥60 % (measure of physical independence).

7- Body mass index (weight/$height^2$) in the range of 18.5 to 30 kg/$m^2$.

8- Normal electrocardiogram recording on a 12-lead electrocardiogram or considered NCS by investigators:
   - 120 < PR <210 ms
   - QRS <120 ms
   - QTcf ≤430 ms for males and <450 ms for females
   - No sign of any trouble of sinusal automatism
   - Or considered NCs by investigators

9- Laboratory parameters within the normal range (hematological and blood chemistry tests). Individual values out of the normal range can be accepted if judged clinically non relevant by the investigator.

10- Having given written informed consent.

11- Covered by Health Insurance System and/or in compliance with the recommendations of National Law in force relating to biomedical research.

*1.2 Exclusion criteria*

1- Presence or history of protein drug hypersensitivity, or allergic disease diagnosed and treated by a physician.

2- Patients who are pregnant or breastfeeding. Patients should not be enrolled if they plan to become pregnant during the time of study participation.

3- Blood donation (including as part of a clinical trial) within 2 months before administration.

4- Patients not able to swallow.

5- Patients with nasogastric tubes.

6- Significant renal disease, defined as a history of chronic renal failure requiring dialysis or kidney transplant, calculated creatinine clearance ≤60 mL/min (Cockcroft-Gault formula).

7- Patients taking valproate.

8- No possibility of contact in case of emergency.

9- Exclusion period of a previous study.

10- Patients who, in the judgment of the Investigator, were likely to be non-compliant or uncooperative during the study, or unable to cooperate because of a language problem or poor mental development.

11- Administrative or legal supervision.

12- Patients receiving 150 mg/m² and not eligible to the 200 mg/m² dose.
