# Supplementary material for: A Multicenter Randomized Bioequivalence Study of a Novel Ready-to-Use Temozolomide Oral Suspension vs. Temozolomide Capsules
Source: Pharmaceutics. 2023 Nov 24;15(12):2664. doi: 10.3390/pharmaceutics15122664 (PMC10747054; doi:10.3390/pharmaceutics15122664)
Supplement: Supplementary file 1 [file pharmaceutics-15-02664-s001.zip › Supplementary S2.pdf]

Supplementary S2*.* Study period flow chart

Visit/Period																	
**Day**	**D-1 (Optional)**	**D1 or D2**															
**Theoritical time**		**Pre-dose**	**T0h**	**T10min**	**T20min**	**T30min**	**T45 min**	**T1h**	**T1h30**	**T2h**	**T2h30**	**T3h**	**T4h**	**T6h**	**T8h**	**T10h**	**T10h+X**
Inclusion/exclusion criteria		X															
Prior/concomitant medications	X																
Physical examination including buccal examination		X															X[c]
Hematology		X[b]															X[c]
Biochemistry		X[b]															X[c]
Pregnancy test (urine)		X[b]															
Admission	X	X															
Discharge																	X[c]
Randomization		X[b]															
Ondansetron premedication		X[d]															
Study Drug Administration			X														
Meals[a]													X				X[b]
12-lead ECG recording																	X[c]
Blood pressure and heart rate		X															X[c]
Blood sample for Pharmacokinetics		X		X	X	X	X	X	X	X	X	X	X	X	X	X	
AE collection	X																

[a]At D1 and D2, patients received a standardized lunch at T4h after the study drug administration. Lunch was given 10 minutes after blood sampling. At D1 only and in case of hospitalization, patients received a standardized dinner after the last blood sampling i.e., 10h to 12h after drug administration; [b]On D1 only; [c]On D2 only; [d]30 minutes before drug administration. D, day; ECG, electrocardiogram.
