# Supplementary material for: A Multicenter Randomized Bioequivalence Study of a Novel Ready-to-Use Temozolomide Oral Suspension vs. Temozolomide Capsules
Source: Pharmaceutics. 2023 Nov 24;15(12):2664. doi: 10.3390/pharmaceutics15122664 (PMC10747054; doi:10.3390/pharmaceutics15122664)
Supplement: Supplementary file 1 [file pharmaceutics-15-02664-s001.zip › Supplementary S3.pdf]

Supplementary S3. Collection of blood samples

Day	Sampling time	Sample N°	Time window (min)
1	Pre-dose (T0)	P00**	
	T0h10min	P01	+/-1
	T0h20min	P02	+/-1
	T0h30min	P03	+/-1
	T0h45min	P04	+/-1
	T1h	P05	+/-3
	T1h30min	P06	+/-3
	T2h	P07	+/-5
	T2h30	P08	+/-5
	T3h	P09	+/-5
	T4h	P10*	+/-5
	T6h	P11	+/-5
	T8h	P12	+/-5
	T10h	P13	+/-5
2	Pre-dose (T0)	P14**	
	T0h10min	P15	+/-1
	T0h20min	P16	+/-1
	T0h30min	P17	+/-1
	T0h45min	P18	+/-1
	T1h	P19	+/-3
	T1h30min	P20	+/-3
	T2h	P21	+/-5
	T2h30	P22	+/-5
	T3h	P23	+/-5
	T4h	P24*	+/-5
	T6h	P25	+/-5
	T8h	P26	+/-5
	T10h	P27	+/-5

*before meal; **sampling before study drug administration.
