# Supplementary material for: A Multicenter Randomized Bioequivalence Study of a Novel Ready-to-Use Temozolomide Oral Suspension vs. Temozolomide Capsules
Source: Pharmaceutics. 2023 Nov 24;15(12):2664. doi: 10.3390/pharmaceutics15122664 (PMC10747054; doi:10.3390/pharmaceutics15122664)
Supplement: Supplementary file 1 [file pharmaceutics-15-02664-s001.zip › Table S1.pdf]

Supplementary Table S1: Medical and surgical history from the ITTS (>10%).

SOC by PT, >10%	Ped-TMZ — TMZ (n=17)			TMZ — Ped-TMZ (n=19)			Overall (N=36)		
	n	%	E	n	%	E	n	%	E
**All SOCs**	**16**	**94.1**	**71**	**14**	**73.7**	**35**	**30**	**83.3**	**105**
**Injury — Poisoning And Procedural Complications**	**2**	**11.8**	**3**	**1**	**5.3**	**2**	**3**	**8.3**	**5**
**Investigations**	**4**	**23.5**	**4**	**1**	**5.3**	**1**	**5**	**13.9**	**5**
Biopsy	3	17.6	3	1	5.3	1	4	11.1	4
**Metabolism And Nutrition Disorders**	**3**	**17.6**	**5**	**0**	**0**	**0**	**3**	**8.3**	**5**
Type 2 Diabetes Mellitus	2	11.8	2	0	0	0	2	5.6	2
**Neoplasms Benign — Malignant And Unspecified**	**3**	**17.6**	**5**	**3**	**15.8**	**4**	6	**16.7**	9
Glioblastoma	3	17.6	3	3	10.5	2	5	13.9	5
**Nervous System Disorders**	**7**	**41.2**	**7**	**0**	**0**	**0**	**7**	**19.4**	**7**
Hemiparesis	2	11.8	2	0	0	0	2	5.6	2
**Psychiatric Disorders**	**3**	**17.6**	**4**	**0**	**0**	**0**	**3**	**8.3**	**3**
Depression	2	11.8	2	0	0	0	2	5.6	2
**Respiratory — Thoracic and Mediastinal Disorders**	2	11.8	3	**0**	0	0	2	5.6	2
**Surgical and Medical Procedures**	**16**	**94.1**	**31**	**11**	**57.9**	**19**	**27**	**75.0**	**50**
Cancer Surgery	1	5.9	1	2	10.5	2	3	8.3	3
Photon Radiation Therapy to Brain	2	11.8	2	0	0	0	2	5.6	2
Radiotherapy	4	23.5	4	3	15.8	3	7	19.4	7
Radiotherapy to Brain	5	29.4	5	4	21.1	4	9	25.0	9
Tumour Ablation	6	35.3	6	7	36.8	7	13	36.1	13
Tumor Escision	4	23.5	6	0	0	0	4	11.1	6
**Vascular Disorders**	**4**	**23.5**	**4**	**2**	**10.5**	**2**	**6**	**16.7**	**6**
Hypertension	4	23.5	4	2	10.5	2	6	16.7	6

%, percentage of patients; E, events; ITTS, intention-to-treat set; n, number of patients; Ped-TMZ, temozolomide oral suspension (KIZFIZO®); PT, Preferred Term; SOC, System Organ Class; TMZ, temozolomide.
