# Supplementary material for: A Multicenter Randomized Bioequivalence Study of a Novel Ready-to-Use Temozolomide Oral Suspension vs. Temozolomide Capsules
Source: Pharmaceutics. 2023 Nov 24;15(12):2664. doi: 10.3390/pharmaceutics15122664 (PMC10747054; doi:10.3390/pharmaceutics15122664)
Supplement: Supplementary file 1 [file pharmaceutics-15-02664-s001.zip › Table S2.pdf]

Supplementary Table S2: Concomitant medications starting before/after TMZ administration from the ITTS (>10%) by treatment sequence and totaled over all patients.

**Concomitant medications,** ATC term >10%	**Ped-TMZ oral suspension — TMZ oral capsule (n=17)**			**TMZ oral capsule — Ped-TMZ oral suspension (n=19)**			**Overall (N=36)**		
	**n**	**%**	**E**	**n**	**%**	**E**	**n**	**%**	**E**
**CLOBAZAM**	2	11.8	2	0	0	0	2	5.6	2
**LACOSAMIDE**	3	17.6	3	2	10.5	2	5	13.9	5
**LAMOTRIGINE**	2	11.8	2	0	0	0	2	5.6	2
**LEVETIRACETAM**	3	17.6	3	8	42.1	8	11	30.6	11
**METFORMINE**	3	17.6	3	0	0	0	3	8.3	3
**PANTOPRAZOLE**	3	17.6	3	1	5.3	1	4	11.1	4
**PARACETAMOL**	2	11.8	2	1	5.3	1	3	8.3	3
**PREDNISOLONE**	7	41.2	7	2	10.5	2	9	25.0	9
**SULFAMETHOXAZOLE; TRIMETHOPRIM**	0	0	0	2	10.5	2	2	5.6	2
**TELMISARTAN**	0	0	0	2	10.5	2	2	5.6	2

%, percentage of patients, ATC, Anatomical Therapeutic Chemical classification; E, events; ITTS, intention-to-treat set; n, number of patients; Ped-TMZ, temozolomide oral suspension (KIZFIZO®); TMZ, temozolomide; TMZ oral capsule, TEMODAL®.
